# Annual acknowledgement of manuscript reviewers

**DOI:** 10.1186/1472-6785-13-3

**Published:** 2013-02-19

**Authors:** Simon Harold

**Affiliations:** 1 BioMed Central, Floor 6, 236 Gray's Inn Road, London WC1X 8HB, UK

## Abstract

**Contributing reviewers:**

The editors of *BMC Ecology* would like to thank all of our reviewers who have contributed to the journal in volume 12 (2012).

## 

Walter Adey

USA

Inger Greve Alsos

Norway

Michel Baguette

France

Yaohui Bai

China

Miklós Bálint

Germany

Peter Banks

Australia

David Baracchi

Italy

Michael Barry

Oman

Kamil Bartoń

Germany

Keith Bayless

USA

Lutz Becks

Germany

Holly Bik

USA

Mark Boyce

Canada

Jason Bragg

Australia

Michael Bull

Austria

Sarah Bush

USA

Luis Cardona

Spain

Antonis Chatzinotas

Germany

Olivier Clerck

Belgium

Ana Cristina Correa

Colombia

Peter Cranston

Australia

Erika Crispo

Canada

Christophe Daugeron

France

Abdoulaye Diabate

USA

Jesse Dillon

USA

Peter Dodson

USA

Frank Drygala

Germany

Vincent Dubut

France

Anne-Béatrice Dufour

France

Mats Dynesius

Sweden

David Erickson

USA

Marie-Anne Felix

France

Ingo Fetzer

Germany

Laura Forrest

UK

Leonard Freed

USA

Lee Frelich

USA

Hélène Fréville

France

Jesus Garcia

Spain

Julien Gasparini

France

Matthew Gilg

USA

Christopher Gobler

USA

Alessandro Grapputo

Italy

Anton Güntsch

Germany

Rob Guralnick

USA

Christiane Hasemann

Germany

Britt Heidinger

UK

Peter Hobbelen

UK

Keith Hobson

Canada

Eric Hoffman

USA

Michael Hofreiter

UK

Robert Holt

USA

Olivier Honnay

Belgium

Diana Huestis

USA

A Randall Hughes

USA

Tine Huyse

Belgium

Kristoffer Hylander

Sweden

Richard Inger

UK

Fumiko Ishihama

Japan

Hans Jacquemyn

Belgium

Mieke Jansen

Belgium

Gerrit Joop

Switzerland

Kurt Jordaens

Belgium

Veijo Jormalainen

Finland

Martyn Kennedy

New Zealand

Tarmo Ketola

Finland

Osamu Kishida

Japan

Kevin Kohl

USA

Jurek Kolasa

Canada

John Kress

USA

Vlastimil Krivan

Czech Republic

Christian Laforsch

Germany

Eric Lamb

Canada

Ramesh Laungani

USA

Daniel Leduc

New Zealand

Charlotte Lee

USA

Alban Lemasson

France

Olivier Lepais

France

Fabien Leprieur

France

Simone Libralato

Italy

Theo Light

USA

Anne Lizé

UK

Carlos Lopez-Vaamonde

France

Beth MacDougall-Shackleton

Canada

Sarina Macfadyen

Australia

Alexei Maklakov

Sweden

Mylene Mariette

France

Rudolf Meier

Singapore

Alexandru Milcu

France

Dmitry Miljutin

Germany

David Miller

USA

William Mitchell

USA

Michael Monaghan

Germany

Juan Manuel Morales

Argentina

Ricardo Moran Lopez

Spain

Peter Morin

USA

Francois Mougeot

Spain

Philip Munday

Australia

Cesc Múrria i Farnós

Spain

Kerstin Musolf

Switzerland

Joan Navarro

Spain

Peter Neuhaus

Canada

Tim Newbold

UK

Yoan Paillet

France

C. E. Timothy Paine

Switzerland

Steve Palmer

UK

Charles Parker

USA

Kevin Pauwels

Belgium

Sandrine Pavoine

France

Thomas Pike

UK

Daniel Pinero

Mexico

Melody Porlier

Canada

Teresita Porter

Canada

Damiano Preatoni

Italy

Benoit Pujol

France

Jessica Purcell

Switzerland

Alex Pyron

USA

Donald Quicke

UK

Ettore Randi

Italy

Björn Rogell

Australia

Sergio Roiloa

Portugal

Lee Rollins

Australia

Davi Rossatto

Brazil

Olivia Roth

Germany

Olivier Roux

Burkina Faso

Marjo Saastamoinen

Finland

Marek Sammul

Estonia

Natalia Sapoukhina

France

Robert Schick

USA

Philip Seddon

New Zealand

Harunobu Shibao

Japan

Tijm Shreeve

UK

Mathieu Sicard

France

Alison Smith

USA

Adrian Smith

USA

Adam Smith

USA

Michael Speed

UK

Felix Sperling

Canada

Dieter Spiteller

Germany

Kelley Thomas

USA

Sunny Townsend

UK

Justin J Travis

UK

Loic Tudesque

France

Evan Twomey

USA

Janske van de Crommenacker

Netherlands

Sebastien Villeger

France

Meghan Vyleta

Austria

Terry Wheeler

Canada

Anja Widdig

Germany

Robert Wisseman

USA

Felix Zajitschek

Sweden

Elizabeth Zimmer

USA

